# On the Use of Cocaine in Genital Irritation in Men

**Published:** 1892-09

**Authors:** 


ON THE USE OF COCAINE IN GENITAL IRRITATION IN MEN.—(*Therapeutic Gazette*, May, 1892.) By H. Wells, M. D. The writer gives a series of cases in which genital irritation in men was much relieved by the injection of about half a drachm of a four per cent. solution of cocaine into the posterior urethra. He recommends the application of the solution locally, in cases of irritation of the glans or prepuce in growing boys, and in the nocturnal incontinence of urine sometimes dependent on such irritation. The effect, according to the writer, lasts for some time. He was led to this use of the drug by noticing that, after the free use of cocaine in nasal and pharyngeal catarrh on all occasions, when he was able to examine it, the penis was much retracted and there was a decided reduction in the sensitiveness of the glans.—*Ex.*

